# Impact of In-Feed versus In-Water Chlortetracycline and Tiamulin Administrations on Fecal Prevalence and Antimicrobial Susceptibilities of *Campylobacter* in a Population of Nursery Pigs

**DOI:** 10.3390/microorganisms11122876

**Published:** 2023-11-28

**Authors:** Victor L. Ishengoma, Raghavendra G. Amachawadi, Mike D. Tokach, Xiaorong Shi, Qing Kang, Robert D. Goodband, Joel DeRouchey, Jason Woodworth, Tiruvoor G. Nagaraja

**Affiliations:** 1 Department of Clinical Sciences, College of Veterinary Medicine, Kansas State University, Manhattan, KS 66506, USA; victorishengoma@gmail.com; 2 Department of Animal Sciences & Industry, College of Agriculture, Kansas State University, Manhattan, KS 66506, USA; mtokach@ksu.edu (M.D.T.); goodband@ksu.edu (R.D.G.); jderouch@ksu.edu (J.D.); jwoodworth@ksu.edu (J.W.); 3 Department of Diagnostic Medicine and Pathobiology, College of Veterinary Medicine, Kansas State University, Manhattan, KS 66506, USA; xshi@vet.ksu.edu (X.S.); tnagaraj@vet.ksu.edu (T.G.N.); 4 Department of Statistics, College of Arts and Sciences, Kansas State University, Manhattan, KS 66506, USA; kangqing@hotmail.com

**Keywords:** piglets, antibiotic administration route, in-feed, in-water, chlortetracycline, tiamulin, *Campylobacter*

## Abstract

Antimicrobial resistance (AMR) in bacteria is a major public health concern in the US and around the world. *Campylobacter* is an important foodborne pathogen that resides in the gut of pigs and is shed in feces, with the potential to be transmitted to humans. In pigs, the oral route, either in-feed or in-water, is by far the most common route of administration of antimicrobials. Because the distribution of the antibiotic in the gut and the dosages are different, the impact of in-feed vs. in-water administration of antibiotics on the development of AMR is likely to be different. Therefore, a study was conducted to compare in-feed vs. in-water administrations of chlortetracycline (CTC) and/or tiamulin on fecal prevalence and AMR profiles of *Campylobacter* among weaned nursery piglets. A total of 1,296 weaned piglets, allocated into 48 pens (27 piglets per pen), were assigned randomly to six treatment groups: Control (no antibiotic), in-feed CTC, in-water CTC, in-feed tiamulin, in-water tiamulin, or in-feed CTC and tiamulin. Fecal samples were collected randomly from 5 piglets from each pen during the pre-treatment (days 0, 7), treatment (days 14, 21), and post-treatment (days 28, 35) phases. Bacterial isolations and species identifications were conducted by culture and PCR, respectively. The microbroth dilution method with Sensititre^TM^ plates was used to determine the antimicrobial susceptibility and resistance of *Campylobacter* isolates. The results on resistance were interpreted based on the European Committee on Antimicrobial Susceptibility Testing (EUCAST) epidemiological cutoff values for *Campylobacter*. The overall prevalence of *Campylobacter* was 18.2% (262/1440). Speciation of *Campylobacter* isolates by PCR indicated the prevalence of only two species: *Campylobacter hyointestinalis* (17.9%; 258/1440) and *C. coli* (0.3%; 4/1440). *Campylobacter* isolates were resistant to tetracycline (98.5%), ciprofloxacin (89.3%), and nalidixic acid (60.3%). Neither the antibiotic nor the route of administration had an effect (*p* > 0.05) on the prevalence of AMR *Campylobacter* in the feces of piglets.

## 1. Introduction

Antimicrobial Resistance (AMR) has emerged as a global threat to human and animal health [1]. In 2020, the World Health Organization declared AMR among the top health challenges in the world [2]. According to the Centers for Disease Control and Prevention (CDC), more than 2.8 million AMR bacterial and fungal infections and 35,900 million deaths occur every year in the United States [3]. There is evidence that AMR infections in humans are linked to the increased and/or frequent use of antimicrobials in animal agriculture [4]. Antimicrobials have been used in swine production on a routine basis as a management tool for many years [5]. According to the United States Food and Drug Administration, approximately six million kilograms of medically important antimicrobials were used for food animal production, with the swine industry utilizing 42% of these antimicrobials [6]. The public health risks associated with the use of antimicrobials in animals have prompted the FDA to ban the use of medically important antimicrobials for growth promotion (FDA Guidance for Industry #209, 2017). The European Centre for Disease Prevention and Control (ECDC), European Food Safety Authority (EFSA), and European Medicines Agency (EMA) have jointly reported a positive association between antimicrobial use in food-producing animals and the AMR of bacteria, including *Campylobacter* [1]. *Campylobacter* is one of the leading causes of foodborne diarrheal illnesses in the US and poses a significant public health threat [7]. The thermophilic *Campylobacter* species are among the pathogens that are becoming increasingly resistant to important and critically important antimicrobials. In 2019, the CDC categorized drug-resistant *Campylobacter* as a serious-level threat. In the US, it is estimated to cause approximately 448,400 infections and about 70 deaths every year [3]. The CDCs National Outbreak Reporting System (NORS) reported a total of 813 *Campylobacter* outbreaks between 2008 and 2021 (https://wwwn.cdc.gov/norsdashboard/; accessed on 27 November 2023). Pigs (and also broiler chickens) are a major reservoir for *Campylobacter* spp., particularly *Campylobacter (C.) coli*, and therefore play a role in transferring the bacteria to human beings [8,9,10,11,12,13]. However, the primary species of *Campylobacter* pathogen in humans is *C. jejuni*, which is very rare in pigs, indicating that pigs are a rare source of human *Campylobacteriosis* [14,15].

Referring to the US, several antimicrobials, including medically important antimicrobials, are used in swine production for therapeutic and non-therapeutic purposes. Tetracyclines and tiamulin are the most commonly used antibiotics in swine [16]. Tetracyclines are widely employed either alone or in combination with other antibiotics for the prevention, control, and treatment of bacterial diseases in swine [17,18]. Tiamulin is extensively used in the swine industry to control and treat dysentery (caused by *Brachyspira hyodysenteriae*), respiratory diseases, and for growth promotion in some countries [19]. Tiamulin is an antibiotic that is used exclusively in swine and poultry and does not require a veterinary feed directive for its usage, which increases the likelihood of being frequently used or even overused by producers. Previous studies have shown that tiamulin and its metabolites select for resistance among *C. coli* [20].

Oral medication is the most commonly used route of antimicrobial administration in pig production [21,22,23]. Several studies have reported oral antimicrobial medication as a driving factor for the development of AMR [24,25,26]. Risk factors such as prolonged antimicrobial use, under-dosing, and environmental contamination with antimicrobials may favor the development of bacterial resistance [26]. Despite a broad array of AMR risks associated with oral route (in-feed and/or in-water) antimicrobial administration, its impact on the amplification and persistence of AMR in *Campylobacter* is a largely unexplored area. We hypothesize that a water-soluble antibiotic, administered in drinking water, is likely to be distributed more uniformly in the gut and, therefore, has a greater impact on the AMR of gut bacteria than the same antibiotic administered in dry form in the feed. Therefore, the objective of this study was to investigate the effect of two different oral administration methods (in-feed vs. in-water) on the AMR profiles of *Campylobacter* spp., shed in the feces of piglets.

## 2. Materials and Methods

### 2.1. Animal and Experimental Design

The Kansas State University Institutional Animal Care and Use Committee approved all experimental protocols intended for this study (IACUC # 4033; Manhattan, KS, USA). Experiments were conducted at a commercial research nursery facility located in Pipestone, Minnesota. A total of 1440 weaned piglets (L337 × 1050, PIC, Hendersonville, TN, USA) were used in a 35-day trial. The piglets were randomly allocated into 48 pens, with each pen containing 27 piglets. The distribution of piglets in pens was made to ensure the average weight between pens was approximately equal. The piglets were provided with 7 days of acclimatization, after which they were randomly assigned to six treatment groups. The treatments included the control (no antibiotic), in-water chlortetracycline (22 mg/kg) BW (CTC) (CTC-hydrochloride, Elanco Animal Health, Indianapolis, IN, USA), in-feed CTC (22 mg/kg) BW, in-water tiamulin (23 mg/kg) BW (Denagard^®^, Elanco, Animal Health, Indianapolis, IN, USA), in-feed tiamulin (5 mg/kg) BW, in-feed CTC (22 mg/kg) BW, and tiamulin (5 mg/kg) BW. The doses for both antibiotics were selected in accordance with legally followed guidelines for their usage. These treatments were applied for 14 days. All 48 pens were used in the trial, allowing for eight replications per treatment.

### 2.2. Fecal Samples

Fresh fecal weekly sample collection was carried out by gentle rectal massage of the first 5 piglets caught out of 27 piglets in the pen (convenience sampling). Fecal samples from each piglet were collected into individual sterile plastic bags (Whirl-Pak^®^ bags, Nasco, Ft. Atkinson, WI, USA) and transported on ice in a cooler to the Pre-harvest Food Safety Laboratory, College of Veterinary Medicine, Kansas State University. Samples were stored at 4 °C and processed within 24 h of collection. 

### 2.3. Isolation of Campylobacter from Fecal Samples

Approximately 1 g of fecal sample was mixed in 9 mL of Mueller-Hinton broth (MH broth; BD Difco, MD, USA) containing two *Campylobacter* selective growth supplements, Oxoid™ *Campylobacter* Growth Supplement (SR0232E) and Oxoid™ Modified Preston *Campylobacter* Selective Supplement (SR0204E) (Thermo Fisher Scientific, Fair Lawn, NJ, USA) (MH+SS agar) in 50 mL filter centrifuge tubes (TubeSpin^®^ Bioreactor, TPP Techno Plastic Products AG, Trasadingen, Switzerland). The tubes were then incubated in a Tri-Gas incubator (BINDER, Tuttlingen, Germany) at 42 °C for 48 h in a microaerophilic atmosphere. A sterile loopful (~10 µL) of the enriched fecal suspension was streaked onto MH+SS agar and incubated in a Tri-Gas incubator at 42 °C for 48 h. Three well-isolated, circular, transparent, and glassy colonies were picked and streaked onto MH agar to confirm growth and obtain pure cultures. Plates were incubated in a Tri-Gas incubator at 42 °C for 48 h in a microaerophilic atmosphere. Pure cultures from the MH agar plate were stored in MH broth with 30% glycerol for future use at −80 °C.

### 2.4. DNA Isolation and Speciation 

DNA was extracted as per the published procedure [27]. Two colonies from MH agar were mixed with 40 µL of single cell lysis buffer (SCLB) (Thermo Fisher Scientific, Fair Lawn, NJ, USA) in a 200 µL polymerase chain reaction (PCR) tube and mixed thoroughly. The SCLB solution contained 1 mL Tris-EDTA (1X TE) (Thermo Fisher Scientific, Fair Lawn, NJ, USA) and 10 µL proteinase K (5 mg/mL) (Sigma-Aldrich, Co., St. Louis, MO, USA). The nuclease-free water was used as a blank for our experiments. To perform cell lysis, the 200 µL PCR tubes were kept in a PCR machine at 80 °C for 10 min and 55 °C for another 10 min. For DNA elution, 40 µL of distilled water was added to the tube, mixed, and centrifuged for 2 min at 4500× *g* to obtain the DNA supernatant.

Speciation of *Campylobacter* was conducted using the multiplex polymerase chain reaction assay [28]. The procedure involved simultaneous detection of 16S rRNA for the genus *Campylobacter*, 23S rRNA for *C. hyointestinalis* subsp. *hyointestinalis*, askD for *C. coli*, *cstA* for *C. fetus*, *glyA* for *C. lari*, *cj0414* for *C. jejuni*, and *lpxA* for *C. upsaliensis*. Primer sequences of the target genes and their amplicon sizes are shown in Table 1. The PCR reaction of 20 µL consisted of 10 µL of master mix (iQ™ Multiplex Powermix-Bio-Rad, Hercules, CA, USA), 5 µL of nuclease-free water, 4 µL of template, and 1 µL of 7 pairs of primers (100 pM) mix (7 pM for each primer). The nuclease-free water was used as a blank for our experiments. Reactions were run on a master-cycler gradient thermal cycler (Eppendorf, Germany), using the following conditions: initial denaturation at 95 °C for 3 min, followed by 25 cycles of denaturation at 95 °C for 30 s, annealing at 61 °C for 90 s, extension at 72 °C for 60 s, and final extension at 72 °C for 7 min. Analysis of PCR products was conducted by capillary gel electrophoresis in a QIAxcel Fast Analyzer system (Qiagen, Valencia, CA, USA).

### 2.5. Antimicrobial Susceptibility Testing

Commercial premade antimicrobial panel plates (Sensititre^TM^ CAMPY, Trek Diagnostic Systems, Thermo Scientific, Oakwood Village, OH, USA) consisting of nine antimicrobials were used to determine the minimal inhibitory concentrations (MICs) of *Campylobacter* isolates. The antimicrobials tested were azithromycin, ciprofloxacin, clindamycin, erythromycin, florfenicol, gentamicin, nalidixic acid, telithromycin, and tetracycline. Two to three single isolated *Campylobacter* colonies were mixed in demineralized water (Trek Diagnostic Systems, Thermo Scientific) to obtain 0.5 McFarland turbidity standards. Subsequently, a 10 µL aliquot of the suspension was transferred to MH broth (Trek Diagnostic Systems, Thermo Scientific) and vortexed. Then, 50 µL of the culture was inoculated into the Sensititre^TM^ plates and incubated in a Tri-Gas incubator under microaerophilic conditions for 24 h at 42 °C. The epidemiological cutoff values established by the European Committee on Antimicrobial Susceptibility Testing (EUCAST) were used to interpret the MIC values as either resistant or susceptible (https://www.eucast.org/fileadmin/src/media/PDFs/EUCAST_files/Breakpoint_tables/v_11.0_Breakpoint_Tables.pdf; accessed on 16 November 2023). 

### 2.6. PCR Detection of Tetracycline Resistance Genes 

A subset of 114 *Campylobacter* isolates [*C. hyointestinalis* (n = 110) and *C. coli* (n = 4)] across all sampling days and treatment groups were subjected to PCR detection of tetracycline resistance genes. *Campylobacter* was tested for the presence of *tetA*, *tetB*, *tetO*, and *tetS* genes using a PCR protocol modified from a published procedure [35]. The genes were screened individually by regular PCR using a Master-cycler gradient thermal cycler (Eppendorf, Germany). The 20 µL reaction mixture consisted of 10 µL master mix, 4 µL nuclease-free water, 4 µL of DNA template, and 1 µL of each forward and reverse primer. The reaction was carried out under the following conditions: 95 °C for 3 min, followed by 30 cycles of denaturation at 94 °C for 60 s, annealing at 56 °C for 60 s, extension at 72 °C for 60 s, and 1 cycle of final extension at 72 °C for 10 min. Information about primers and amplicon sizes is shown in Table 2. Analysis of PCR products was carried out by capillary gel electrophoresis in the QIAxcel Advanced system (Qiagen, Germantown, MD, USA). Tetracycline-resistant *E. coli* strains used as positive controls were acquired from the USDA (Meat Animal Research Center, Clay Center, NE, USA) and Dr. Marilyn C. Roberts at the University of Washington.

### 2.7. Statistical Analysis

Prevalence data were organized as a binomial response at the animal level per pen, i.e., the number of ‘+’ out of 5 samples per pen per sampling day. Binomial data were summarized by species and treatment at each sampling day. Due to the low prevalence of *C. coli*, only *C. hyointestinalis* and the overall *Campylobacter* prevalence were subjected to statistical analysis. The prevalence data were analyzed using the PROC GLIMMIX procedure in SAS (version 9.4; Cary, NC, USA). The binomial response post-treatment was analyzed under the logit linear mixed model with repeated measurements over time. The fixed effects of the model included treatment, sampling day, and their interactions [37,38]. Random effects of the model included blocks and pens. The random pen effect corresponded to the model intercept term and accounted for the correlation of binomial responses from the same pen. Prevalence on sampling day 0 (i.e., baseline response) served as the model covariate. All tests were conducted at the 0.05 significance level. A comparison between two levels of a model fixed effect was carried out using a 2-sided test. Distributions of test statistics were approximated by Chi-square distributions. Treatment groups were compared within and across sampling days due to no differences observed between treatment and sampling day interactions.

The MIC data were summarized by species and treatment at each sampling day, and analysis was performed using the SAS PROC MIXED procedure. Due to the limited number of isolates tested for MIC, *C. coli* data at sampling day 0 and *C. hyointestinalis* data at sampling day 7 were excluded from the statistical analysis. The MIC data of *C. hyointestinalis* were analyzed separately at every post-treatment sampling day under the linear model, with treatment being the fixed effect. To better achieve model assumptions, data underwent natural log transformation before statistical modeling. The treatment effect was assessed via back-transformed least squares (LS) means and mean differences. Comparisons were carried out using the 2-sided test. 

## 3. Results

### 3.1. Prevalence of Campylobacter

A total of 262 (18.2%) *Campylobacter* isolates were recovered from 1440 samples. The majority of these were *C. hyointestinalis* (258; 17.9%) and (4; 0.3%). *C. coli*. The prevalence distributions across different treatments, treatment phases, and sampling days are summarized in Table 3. The prevalence of *Campylobacter* was similar between in-feed tiamulin (53; 22.1%), in-feed CTC (52; 21.7%), and control (49; 20.4%) when compared to in-water CTC (37; 15.4%), in-feed CTC + tiamulin combination (37; 15.4%), and in-water tiamulin (34; 14.2%) treatment groups. Based on treatment phases, the post-treatment phase had a numerically higher number of isolates (168; 35%) when compared to 75 (15.6%) and 19 (4.0%) in the treatment and pre-treatment phases, respectively. The four *C. coli* isolates were all isolated in the pre-treatment phase. Overall, there was no significant effect of treatment on the prevalence of *Campylobacter* (*p* > 0.05) (Table 4), although the sampling day effect was significant (*p* < 0.05). Also, there was no sampling day and treatment interaction for the prevalence of *Campylobacter* (*p* > 0.05) (Table 5). 

### 3.2. Antimicrobial Susceptibilities

The MIC distributions and resistance percentages of *Campylobacter* isolates (n = 262) against the nine antimicrobials used are depicted in Table 6. The *Campylobacter* isolates (n = 262) were highly resistant to tetracycline (98.5%), ciprofloxacin (89.3%), and nalidixic acid (60.3%). Low resistance to gentamicin (8%), azithromycin (5%), erythromycin (3.4%), clindamycin (3.1%), telithromycin (1.9%), and florfenicol (8%) was also observed in this study. A total of six different resistance profiles were observed in this study (Table 6). The most common resistance profile was CIP_NAL_TET, which was observed in 122 (46.6%) isolates, followed by CIP_TET in 83 (31.7%), CIP_GEN_NAL_TET, and NAL_TET in 14 (5.3%) isolates, with 8 (3.1%) isolates having resistance to tetracycline alone. A total of 31 (11.8%, n = 262) isolates were resistant to more than three antimicrobial classes, thus recording multidrug resistance. Four of these isolates exhibited multidrug resistance to five or more antimicrobial classes, with the highest resistance shown by two strains to seven antimicrobial classes. Three of the isolates were resistant to four antimicrobial classes, and the rest of the 24 isolates were resistant to three antimicrobial classes.

### 3.3. Prevalence of Tetracycline Resistance Genes

*Campylobacter* isolates (n = 114) were screened for the presence of tetracycline resistance genes (*tet*A, *tet*B, *tet*O, and *tet*S) by PCR. All 114 strains were positive for the *tet*O gene. The *tet*A, *tet*B, and *tet*S genes were not detected in these strains.

## 4. Discussion

Swine are considered significant reservoirs of *Campylobacter,* particularly *C. coli* and *C. hyointestinalis*, as the predominant species [14,15,39]. Species such as *C. coli*, *C. jejuni*, *C. lari*, and *C. hyointestinalis*, which are linked to gastrointestinal illness in humans, hold significant public health significance [15]. According to the CDC, *Campylobacter* is one of the four major causative agents of foodborne gastroenteritis in the US (https://www.cdc.gov/foodsafety/foodborne-germs.html; accessed on 16 November 2023) with foods of animal origin being the most common source. Moreover, there is a growing concern as *Campylobacter* spp., are increasingly developing resistance to antimicrobials employed in treating the infections, thereby presenting an additional threat to public health [40,41,42]. 

Several studies have reported the use of antimicrobials in conventional swine production farms in the US [43,44]. Tetracycline and tiamulin are among the most commonly used antimicrobials in swine production [18,20]. Tetracyclines are the leading antimicrobial class used in all production stages of swine in the US and globally [20,45]. Tiamulin is commonly used in the treatment of diseases in swine, especially weaners and growers [46]. The use of antimicrobials, including tetracycline and tiamulin, is associated with AMR in *Campylobacter* [44,47]. A study by Quintana-Hayashi et al. [48] recorded an association between the use of tetracyclines and the AMR of *Campylobacter* in nursery piglets. Similarly, one study observed that tiamulin metabolites select for AMR in *C. coli* [21]. 

The overall animal-level prevalence of *Campylobacter* in this study was 18.2% (262/1440). A study among piglets reported a prevalence of 27.6% in conventional farms as compared to 77.3% in antibiotic-free farms [43]. But this prevalence is relatively lower than what has been reported in grower pigs [49,50], in contrast to [51] findings that nursery piglets were more likely to get colonized with *Campylobacter* spp. than sows. The majority of the isolates in our study were *C. hyointestinalis*, 17.9% (258/1440), and *C. coli*, only 0.3% (4/1440). Indeed, *C. hyointestinalis* is commonly found in swine [52]. However, most studies conducted so far have primarily focused on *C. jejuni* and *C. coli* in swine. Nevertheless, there is a consensus that *C. hyointestinalis* is an emerging pathogen responsible for gastrointestinal illness in humans, underscoring its significance [53,54]. The low prevalence of *C. coli* recorded in this study was surprising. Many studies have shown *C. coli* to be the most prevalent *Campylobacter* spp., in swine [8,14,49,51,55]. *C. coli* and *C. jejuni* are both known to be associated with causing gastroenteritis in humans [49,54]. 

*Campylobacter* isolates were highly resistant to the critically important class of antimicrobials, the fluoroquinolones (ciprofloxacin) and quinolones (nalidixic acid). Similar results were recorded in other studies that have reported high resistance to ciprofloxacin and nalidixic acid [43,56]. In addition, the majority of *Campylobacter* isolates were resistant to a medically important antimicrobial, tetracycline. The presence of *tetO* in all the isolates tested accounts for the high resistance to tetracyclines (98.5%) observed in this study. The *tetO* gene is both chromosomally and plasmid-associated and can be transferred between bacteria of the same and different species [57]. The ability of the *tetO* gene to be transferred intraspecies and interspecies explains the high prevalence of *Campylobacter* species reported [10].

In this study, we evaluated the impact of in-feed versus in-water antimicrobial administration and non-treated controls on the fecal prevalence and antimicrobial susceptibilities of a foodborne pathogen, *Campylobacter*, in nursery piglets. We initially hypothesized that the oral route of administering antimicrobials has a higher impact on *Campylobacter* prevalence and resistance to antimicrobials. Indeed, increased resistance in the gut microbiota was observed when mice were orally treated with antibiotics compared to cases where parenteral routes were utilized [58]. In our study, neither in-feed nor in-water routes of antibiotic administration had a significant impact on the prevalence and AMR of *Campylobacter* compared to untreated controls. Similarly, treating piglets with either tiamulin or tetracycline or the combination of both did not significantly influence the prevalence and AMR of *Campylobacter*. Similar results were recorded by a study in which tetracycline administration in pigs was not associated with a change in the resistance of *Campylobacter* to tetracycline, ciprofloxacin, and erythromycin [55]. High *Campylobacter* resistances have been recorded even in antibiotic-free farms, indicating that the development of resistance to the pathogen does not necessarily result from antimicrobial usage [44,48].

In contrast, an increased tetracycline resistance of *Campylobacter* spp. following tetracycline treatment in animals was reported [50]. The speculation is that the lack of distinction between treatment groups may be attributed to either an influence of the environment or the stabilization of the microbiota in response to the presence of tiamulin and tetracycline. However, this was not investigated in this study. 

The effect exerted by antimicrobials on gut pathogens could be influenced by the dosage used, distribution, and their ultimate concentration in the gut [59,60,61,62]. In this study, we used the dosages as per FDA guidance for the treatment or prevention of diseases [63]. The same dosage of chlortetracycline, 22 mg/kg BW, was provided in both in-feed and in-water administration, while for tiamulin, a 23 mg/kg BW dosage was applied in-feed and 5 mg/kg BW in-water. Furthermore, there exists a linear relationship between the dosage of antimicrobials and their concentrations in the gut t [64]. However, the dose of a drug is indirectly proportional to its bioavailability [65]. Hence, the comparable gut concentrations of tetracycline and tiamulin in both in-feed and in-water may account for the observed differences in the prevalence and AMR of bacteria. In addition, the two drugs, chlortetracycline and tiamulin, have enough concentrations in the gut, which explains their use in treating enteric infections [66]. Tetracyclines have low bioavailability, making a large amount of the drug remain in the gut and exerting its effects on gut bacteria, as documented in [66]. On the other end, tiamulin has high bioavailability and is metabolized quickly and excreted in the gut via bile to exert its effect on the gut microorganisms [67]. This implies that the impact of tiamulin might be more significant due to its higher bioavailability compared to tetracycline.

In summary, the route of administration of CTC and tiamulin did not significantly impact the prevalence and AMR of *Campylobacter* in the feces of piglets. In contrast to what is reported in many studies, *C. hyointestinalis*, and not *C. coli*, was the dominant species in the feces of piglets. A large number of *Campylobacter* strains were multidrug resistant and exhibited a wide range of resistance profiles that included resistance to a fluoroquinolone (ciprofloxacin), which is frequently used to treat *Campylobacter* infections. Because of the high prevalence of CTC resistance in *Campylobacter* in the herd, the impact of the route of administration could not be measured. Our research findings support pork producers as they seek to improve their herds’ economic health and promote the welfare of pigs and public health.

## Figures and Tables

**Table 1:** Gene targets, primer sequences, and amplicon sizes were used for the speciation of *Campylobacter* isolated from piglets administered with in-feed or in-water chlortetracycline (CTC) and/or tiamulin.

Species	Primer	Target Gene	Sequence (5′ to 3′)	Amplicon Size	Reference
Genus *Campylobacter*	C412F C1228R	16S rRNA	5′-GGATGACACTTTTCGGAGC-3′ 5′-CATTGTAGCACGTGTGTC-3′	816	[29]
*C. hyointestinalis* subsp. *hyointestinalis*	HYO1F HYOFET23SR	23S rRNA	5′-ATAATCTAGGTGAGAATCCTAG-3′ 5′-GCTTCGCATAGCTAACAT-3′	611	[30]
*C. coli*	CC18F CC519R	*askD*	5′-GGTATGATTTCTACAAAGCGAG-3′ 5′-ATAAAAGACTATCGTCGCGTG-3′	502	[31]
*C. fetus*	MG3F CF359R	*cstA*	5′-GGTAGCCGCAGCTGCTAAGAT-3′ 5′-AGCCAGTAACGCATATTATAGTAG-3′	359	[32]
*C. lari*	CLF CLR	*glyA*	5′-TAGAGAGATAGCAAAAGAGA-3′ 5′-TACACATAATAATCCCACCC-3′	251	[33]
*C. jejuni*	C-1 C-3	*cj0414*	5′-CAAATAAAGTTAGAGGTAGAATGT-3′ 5′-CCATAAGCACTAGCTAGCTGAT-3′	161	[34]
*C. upsaliensis*	CU61F CU146R	*lpxA*	5′-CGATGATGTGCAAATTGAAGC-3′ 5′-TTCTAGCCCCTTGCTTGATG-3′	86	[28]

**Table 2:** Target genes, primer sequences, and amplicon sizes used for detection of *tet* (A, B, O, and S) resistance genes in *Campylobacter* isolated from piglets administered with in-feed or in-water chlortetracycline (CTC) and/or tiamulin.

Primer	Target Gene	Sequence (5′ to 3′)	Amplicon (bp)	Reference
*tetA*-F	*tet*(A)	GTGAAACCCAACATACCCC	888	[35,36]
*tetA*-R		GAAGGCAAGCAGGATGTAG		
*tetB*-F	*tet*(B)	CCTTATCATGCCAGTCTTGC	774	[35,36]
*tetB*-R		ACTGCCGTTTTTTCGCC		
*tetS*-F	*tet*(S)	CATAGACAAGCCGTTGACC	667	[35,36]
*tetS*-R		ATG TTT TTG GAA CGC CAG AG		
*tetO*-F *tetO*-R	*tet*(O)	AACTTAGGCATTCTGGCTCAC TCCCACTGTTCCATATCGTCA	515	[35,36]

**Table 3:** Animal-level fecal prevalence of *Campylobacter* in piglets administered with in-feed or in-water chlortetracycline (CTC) and/or tiamulin (n = 1440).

Treatment Groups	Treatment Phases						
	Pre-Treatment		Treatment		Post-Treatment		Treatment Total (%)
	Week 1	Week 2	Week 3	Week 4	Week 5	Week 6	
Control	1	2	8	9	15	13	49 (20.4)
In-feed CTC	0	5	6	12	11	18	52 (21.7)
In-water CTC	2	3	4	3	12	14	37 (15.4)
In-feed Tiamulin	0	1	5	11	19	17	53 (22.1)
In-water Tiamulin	0	2	3	2	14	13	34 (14.2)
In-Feed CTC + Tiamulin	0	3	5	7	13	9	37 (15.4)
**Weekly Total (%)**	3 (1.3)	16 (6.7)	31 (12.9)	44 (18.3)	84 (35.0)	84 (35.0)	
**Total (%)**	19 (4.0)		75 (15.6)		168 (35)		262 (18.2)

**Table 4:** Treatment effects on the fecal prevalence (main effects) of *Campylobacter* in piglets administered with in-feed or in-water chlortetracycline (CTC) and/or tiamulin (n = 1440).

			Comparison to Control
Endpoint	Treatment	Prevalence +/− S.E.	Odds Ratio (*p*-Value for Testing Odds Ratio = 1)
*Campylobacter*	Control	0.274 +/− 0.048	-
(n = 262)	In-feed CTC	0.273 +/− 0.049	0.99 (0.987)
	In-water CTC	0.172 +/− 0.04	0.55 (0.105)
	In-feed Tiamulin	0.306 +/− 0.052	1.16 (0.655)
	In-water Tiamulin	0.153 +/− 0.04	0.48 (0.057)
	In-feed CTC + Tiamulin	0.199 +/− 0.041	0.66 (0.235)
*C. hyointestinalis*	Control	0.275 +/− 0.047	-
(n = 258)	In-feed CTC	0.268 +/− 0.049	0.97 (0.923)
	In-water CTC	0.174 +/− 0.04	0.56 (0.109)
	In-feed Tiamulin	0.308 +/− 0.052	1.17 (0.639)
	In-water Tiamulin	0.153 +/− 0.04	0.48 (0.056)
	In-feed CTC + Tiamulin	0.198 +/− 0.041	0.65 (0.221)

**Table 5:** Interaction effect of treatment vs. sampling day on the prevalence of *Campylobacter* in piglets administered with in-feed or in-water chlortetracycline (CTC) and/or tiamulin (n = 1440).

Endpoint	Treatment	Sampling Day	Prevalence +/− S.E.	Odds Ratio (*p*-Value for Testing Odds Ratio = 1) Comparison to		
				Day 14	Day 21	Day 28
*Campylobacter*	Control	Day 14	0.198 +/− 0.068	0.86 (0.784)	0.41 (0.086)	0.52 (0.205)
(n = 262)		Day 21	0.223 +/− 0.071	--	0.48 (0.144)	0.60 (0.316)
		Day 28	0.375 +/− 0.085	--	--	1.25 (0.637)
		Day 35	0.324 +/− 0.082	--	--	--
	In-feed CTC	Day 14	0.143 +/− 0.058	0.41 (0.111)	0.46 (0.173)	0.21 (0.004)
		Day 21	0.292 +/− 0.08	--	1.13 (0.803)	0.52 (0.164)
		Day 28	0.267 +/− 0.077	--	--	0.46 (0.103)
		Day 35	0.444 +/− 0.089	--	--	--
	In-water CTC	Day 14	0.097 +/− 0.048	1.37 (0.692)	0.26 (0.031)	0.20 (0.011)
		Day 21	0.073 +/− 0.042	--	0.19 (0.016)	0.15 (0.005)
		Day 28	0.296 +/− 0.079	--	--	0.79 (0.630)
		Day 35	0.347 +/− 0.083	--	--	--
	In-feed Tiamulin	Day 14	0.125 +/− 0.055	0.37 (0.100)	0.16 (0.001)	0.19 (0.004)
		Day 21	0.276 +/− 0.078	--	0.42 (0.066)	0.51 (0.160)
		Day 28	0.479 +/− 0.089	--	--	1.23 (0.651)
		Day 35	0.428 +/− 0.088	--	--	--
	In-water Tiamulin	Day 14	0.074 +/− 0.043	1.54 (0.646)	0.15 (0.006)	0.17 (0.009)
		Day 21	0.049 +/− 0.035	--	0.10 (0.003)	0.11 (0.005)
		Day 28	0.35 +/− 0.084	--	--	1.12 (0.812)
		Day 35	0.324 +/− 0.082	--	--	--
	In-feed CTC + Tiamulin	Day 14	0.122 +/− 0.054	0.67 (0.531)	0.29 (0.037)	0.49 (0.242)
		Day 21	0.171 +/− 0.063	--	0.44 (0.123)	0.73 (0.575)
		Day 28	0.322 +/− 0.082	--	--	1.67 (0.315)
		Day 35	0.221 +/− 0.071	--	--	--
*C. hyointestinalis*	Control	Day 14	0.199 +/− 0.068	0.86 (0.784)	0.41 (0.086)	0.52 (0.205)
(n = 258)		Day 21	0.224 +/− 0.071	--	0.48 (0.144)	0.60 (0.316)
		Day 28	0.375 +/− 0.085	--	--	1.25 (0.638)
		Day 35	0.325 +/− 0.082	--	--	--
	In-feed CTC	Day 14	0.14 +/− 0.057	0.40 (0.110)	0.46 (0.173)	0.21 (0.004)
		Day 21	0.287 +/− 0.079	--	1.13 (0.803)	0.52 (0.163)
		Day 28	0.262 +/− 0.076	--	--	0.46 (0.102)
		Day 35	0.438 +/− 0.089	--	--	--
	In-water CTC	Day 14	0.098 +/− 0.049	1.37 (0.692)	0.26 (0.031)	0.20 (0.011)
		Day 21	0.073 +/− 0.042	--	0.19 (0.016)	0.15 (0.005)
		Day 28	0.299 +/− 0.08	--	--	0.79 (0.631)
		Day 35	0.349 +/− 0.084	--	--	--
	In-feed Tiamulin	Day 14	0.126 +/− 0.056	0.37 (0.100)	0.16 (0.001)	0.19 (0.004)
		Day 21	0.278 +/− 0.078	--	0.42 (0.066)	0.51 (0.160)
		Day 28	0.481 +/− 0.088	--	--	1.23 (0.651)
		Day 35	0.431 +/− 0.088	--	--	--
	In-water Tiamulin	Day 14	0.074 +/− 0.043	1.54 (0.646)	0.15 (0.006)	0.17 (0.009)
		Day 21	0.049 +/− 0.035	--	0.10 (0.003)	0.11 (0.005)
		Day 28	0.35 +/− 0.083	--	--	1.12 (0.812)
		Day 35	0.325 +/− 0.082	--	--	--
	In-feed CTC + Tiamulin	Day 14	0.121 +/− 0.054	0.67 (0.531)	0.29 (0.037)	0.49 (0.242)
		Day 21	0.17 +/− 0.063	--	0.44 (0.123)	0.73 (0.574)
		Day 28	0.32 +/− 0.081	--	--	1.67 (0.315)
		Day 35	0.199 +/− 0.068	0.86 (0.784)	0.41 (0.086)	0.52 (0.205)

**Table 6:** Prevalence and MIC* distributions of *Campylobacter* isolates in piglets administered with in-feed or in-water chlortetracycline (CTC) and/or tiamulin (n = 262).

Antimicrobials	Resistant Breakpoints	% Resistant	0.015	0.03	0.06	0.12	0.25	0.5	1	2	4	8	16	32	64	128	256
Azithromycin	≥1	5	11	13	197	23	3	3	0	1	2	3	2	1	3		
Ciprofloxacin	≥1	89.3		1	0	11	10	6	2	4	8	204	16				
Clindamycin	≥2	3.1		28	10	44	163	4	5	1	1	3	3				
Erythromycin	≥16	3.4		22	10	9	39	153	16	4	1	1	1	1	4		
Florfenicol	≥8	1.2				1	18	213	22	4	1	1	1	1			
Gentamicin	≥4	8				28	9	25	109	70	4	2	1	1			
Nalidixic Acid	≥32	60.3										5	99	86	72		
Telithromycin	≥8	1.9	26	4	7	1	8	152	52	7	1	4					
Tetracycline	≥4	98.5					1	1	1	1	4	14	75	86	79		

MIC* = Minimal inhibitory concentration; Note: The top dilution tested for each drug should be interpreted as ≥ and the lowest dilution tested for each drug as ≤.

## Data Availability

The data presented in this study are available in this article.
